# Simultaneous early surgical repair of post-cholecystectomy major bile duct injury and complex abdominal evisceration: A case report

**DOI:** 10.1016/j.ijscr.2022.107110

**Published:** 2022-04-21

**Authors:** Alfredo Torretta, Dimana Kaludova, Mayank Roy, Satya Bhattacharya, Roberto Valente

**Affiliations:** a Department of General Surgery, “Val Vibrata” Hospital, ASL Teramo, Italy; b HPB Surgery Service, Barts and the London Centre, Barts Health NHS Trust, London, UK; c Department of Surgery and Interventional Science, University College London, UK; d Department of Surgery, Ospedale Policlinico San Martino Genova, Italy

**Keywords:** Laparoscopic cholecystectomy bile duct injury, Burst abdomen, Evisceration, Open abdomen, Negative pressure wound therapy (NPWT), Vacuum assisted wound closure and mesh-mediated fascial traction (VAWCM)

## Abstract

**Background:**

Major bile duct injuries (BDIs) are hazardous complications during 0.4%–0.6% of laparoscopic cholecystectomies. Major BDIs usually require surgical repair, ideally either immediately or at least six weeks after the damage. The complexity of our case lies in the coexistence of early BDI followed by 2-week biliary peritonitis with massive midline evisceration which, in combination, has over 40% mortality risk.

**Methods & case report:**

We describe the case of a 65-year-old male, transferred to our tertiary HPB service on day 14 after common bile duct complete transection during cholecystectomy and postoperative laparotomy. The patient presented with biliary peritonitis along with full wound dehiscence and extensive evisceration. During emergency peritoneal wash-out surgery we deemed immediate BDI repair feasible by primary Roux-en-Y hepaticojejunostomy (HJ), with multi-stage abdominal closure. In the following days we performed progressive abdominal wall closure in multiple sessions under general anesthesia, aided by vacuum-assisted wound closure and intraperitoneal mesh-mediated fascial traction-approximation (VAWCM) with permeable mesh. An expected late incisional hernia was eventually repaired through component separation and biological mesh.

**Discussion & conclusion:**

The simultaneous use of Roux-en-Y HJ and VAWCM has proven safe and effective in the treatment of BDI and 2-week biliary peritonitis with massive midline evisceration.

## Highlights

• Bile Duct Injuries (BDIs) are hazardous complications associated to 0.4%-0.6% laparoscopic cholecystectomies.
• Major BDIs usually require surgery, ideally either immediately or at least six weeks after index surgery.
• In this case a major BDI was associated with biliary peritonitis and abdominal evisceration, making high risk operative management necessary.
• Primary Roux-en-Y hepaticojejunostomy combined with contemporary management of the defect of abdominal wall with VAWCM has proven safe and successful.

## 1 Background

### 1.1 Bile duct iatrogenic injuries

Laparoscopic cholecystectomy (LC) is the standard surgical treatment for symptomatic gallstone disease, and one of the most performed abdominal operations by general surgeons [1], [2]. However, iatrogenic bile duct injuries (BDIs) remain a major concern [2], [3], [4]. BDIs are commonly classified as early (when discovered within two weeks after surgery) and late if from the third week on [5]. Their reported incidence in the era of laparoscopic cholecystectomy is higher than in previous open cholecystectomy series (0.4%–0.6% vs. 0.1%–0.2%, respectively) [1], [6], [7], [8]. BDIs are associated to 2–4% mortality rates [5] and figures are expectedly higher if associated with bowel [9], [10], [11] or vascular injuries [9], [10], [11]. Additionally, BDIs determine a decrease in patients' quality of life [9], [10], [11] and often trigger legal claims to surgeons and hospitals [9], [10], [11]. For these reasons, they often require complex decision-making.

Current recommendations include BDI repair either immediately or at least six weeks after cholecystectomy [1], [12], [13]. In our case, we faced the issue fourteen days after cholecystectomy, and we considered operating on the bile leak necessary due to the coexistence of massive burst abdomen requiring immediate repair [14]. However, we were expecting additional technical complexity and postoperative risks related to the BDI damage and repair.

Roux-en-Y Hepaticojejunostomy (HJ) is the most common surgical treatment for major BDIs [15]. Despite its proven relative safety, HJ is burdened by short term morbidity reaching 20–40% [16] and may result in long term complications such as anastomotic stricture in 2–25% [17] with marked affection of quality of life. Our patient presented with full transection of the main bile duct, high-volume biliary spillage in the surrounding peritoneal cavity, only partially cleared by surgical drain in the context of an established diffused biliary subacute peritonitis.

### 1.2 Acute postoperative open abdominal wall

Acute postoperative open abdominal wall (POAW) [18] consists of the separation of the cutaneous, muscular, and aponeurotic layers of the abdominal wall and may be considered as a unique clinical entity resulting from unintentional or intentional surgical-related actions. Unintentional acute POAW corresponds to burst abdomen (also known as acute wound failure, evisceration, wound dehiscence, wound disruption and fascial dehiscence) and is a postoperative complication after primary closure of an abdominal laparotomy incision. Intentional acute POAW is the result of a deliberate therapeutic procedure, the so called “open abdomen”, where an abdominal wall defect is created intentionally leaving the abdominal incision open at the completion of abdominal surgery [18].

Firstly used in trauma care in the 1990s as part of a damage control surgery [19], OA has also been adopted as a lifesaving strategy in patients with severe abdominal sepsis and complex abdominal conditions including burst abdomen [20], [21].

OA technique can be associated with significant complications and should be used carefully only in selected patients [14]. Nowadays the main indications for an OA approach can be summarized into the following:

- visceral edema and/or intraabdominal swelling with reduced intraabdominal space, making the abdomen mechanically impossible to close;
- intraabdominal deep infection/peritonitis needing active drainage;
- damage control and/or planned second look operations;
- decompression of abdominal hypertension or compartment syndrome [22].

We present a case of major BDI associated to burst abdomen through the full midline abdominal wound with complete loss of domain. We also discuss the strategy and the techniques we have utilized based on a brief literature review. This case report was drafted and submitted in line with the SCARE guidelines [56].

## 2 Case presentation

A 65-year-old male with type 2 diabetes and otherwise unremarkable past medical history underwent laparoscopic cholecystectomy for symptomatic gallstones in a general district hospital. He was discharged on the same day, following apparently uneventful surgery. He however represented to the Emergency Department two days later with nausea vomiting, abdominal pain and fever. On examination he was febrile with a temperature above 38 °C, a blood pressure of 110/80 mm Hg and a tachycardia. His abdomen was swollen and tender with Blumberg sign. Laboratory findings were consistent with biliary peritonitis, including elevated white blood cell count and C-reactive protein. Liver function tests showed elevated bilirubin, cholestatis and hepatocyte necrosis enzymes. An urgent abdominal CT revealed perihepatic and further abdominal collections, and intestinal air-fluid levels.

An Emergency endoscopic retrograde cholangiopancreatography (ERCP) showed full CBD transection (‘E1’ BDI according to Strasberg classification) [3]. The patient was given broad spectrum intravenous antibiotics (IV cephalosporins) to treat sepsis, fluid resuscitation and promptly prepared for open laparotomy.

**Fig. 1:**
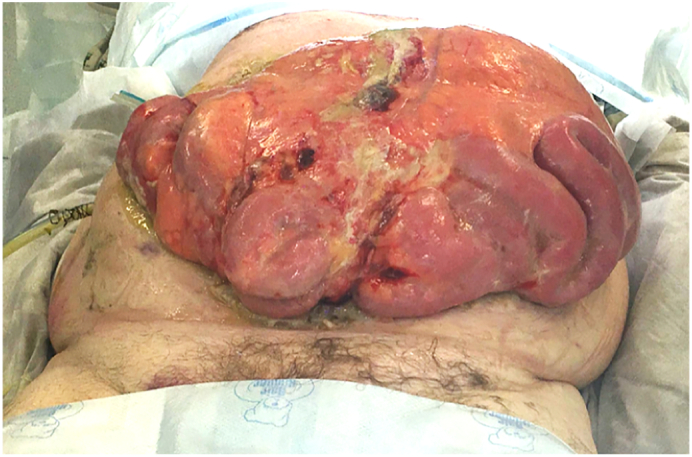
Extensive burst abdomen with loss of domain.

The local surgical team performed midline exploratory laparotomy, washout, and external drainage placing a large-bore tube aside the leaking CBD and referred the case to our tertiary HPB service. While awaiting transfer due to bed shortage, on post-operative day 11 after the laparoscopic cholecystectomy a wound dehiscence was locally treated by vacuum therapy dressing, eventually stopped due to full bowel herniation. At transfer arrival at our HPB service on postoperative day 14 after index cholecystectomy, the patient presented extensive burst abdomen with loss of domain and persisting high-volume biliary spillage in between edematous mesentery and bowels (Fig. 1). Surprisingly, he was asymptomatic, without clinical manifestations of sepsis presumably due to a full and spontaneous external biliary drainage allowed by the complete evisceration, prevented the development of biliary peritonitis or abdominal collections.

Following patient assessment, literature fast review and informed consent, we scheduled urgent surgery and planned multi-stage operations, as the abdominal wall was obviously impossible to close in a single session, likely requiring OA technique.

At surgery, after adhesiolysis and washout, we identified the fully transected main bile duct. Given the absence of major vascular injury, after trimming its edge it showed trophic wall, allowing primary hepaticojejunostomy (HJ) by an interrupted 4/0 PDS suture.

**Fig. 2:**
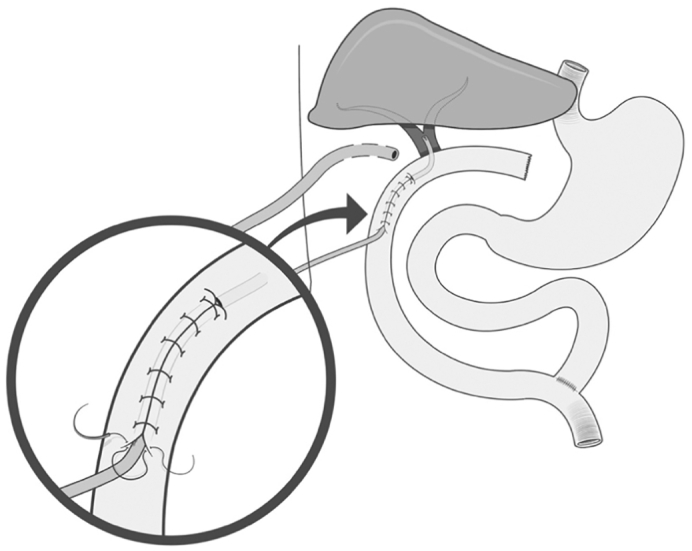
Hepaticojejunostomy: Roux-en-Y transmesocolic loop over a feeding tube through a Witzel tunnel.

This was performed by a Roux-en-Y transmesocolic loop over 10F transmural and transcutaneous feeding tube through a Witzel tunnel (Fig. 2).

After completing HJ, reapproximation of the muscular layer of the abdominal wall was as expected impossible due to the established lateral retraction of the rectus muscle following the prolonged omental and bowel herniation, lasting several days. Hence, we opted to repair the burst abdomen by progressive abdominal wall closure in multiple theatre sessions, using the ‘vacuum assisted closure and mesh-mediated fascial traction’ (VAWCM) technique [23]. At first, we utilized an intra- abdominal ‘sandwich’ drape-gauze dressing, as described by Schein [24] and Brock [25]. An Opsite® transparent adhesive dressing was folded over a large flat abdominal gauze pack with both sides folded inwards creating a rectangular configuration in the longitudinal axis (approximately 45 × 45 cm). Both Opsite® drape layers were sharp cut as creating a thin, ‘tally frame’ fenestration on both surfaces, to adsorb and drain intra-abdominal fluids. We placed the drape-gauze ‘sandwich’ dressing on top of the abdominal viscera, with the edges tucked under the abdominal wall, reaching the lateral peritoneal gutters.

**Fig. 3:**
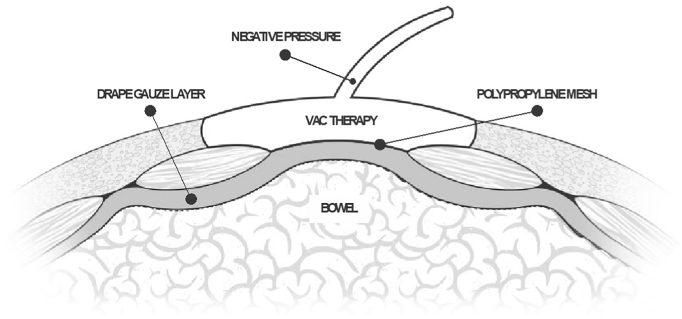
VAWCM technique.

The abdominal wall was then closed by an in-lay polypropylene net mesh, anchored to the lateral fascial edges over a running nylon suture (Fig. 3).

Such mesh was chosen to allow fluid migration through, planning its early removal after abdominal biliary/septic clearance.

**Fig. 4:**
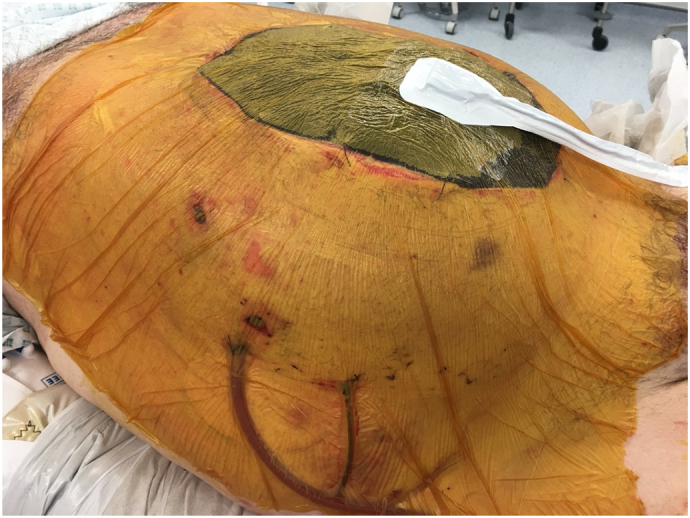
VAC dressing.

Finally, a VAC dressing was applied over the mesh at low pressure (40 mm Hg) (Fig. 4).

At the end of our first operation the patient was stable but was kept intubated and transferred to intensive care unit (ICU) to receive more comprehensive therapies including intravenous antibiotics, fluids, and low-weight heparin. In the immediate postoperative course nutrition was provided via total parenteral nutrition (TPN) while an early enteral nutrition (EN) was started via naso-jejunal feeding tube (25–30 kcal/kg/day). The adequacy of nutrition was estimated monitoring the nitrogen balance, serum total proteins, albumin and transferrin. Some additional 25–30% extra calories above the estimated needs were ensured.

Three days after our first surgery, the patient underwent a second-look laparotomy. Following the removal of the VAC dressing, we incised the polypropylene (PP) mesh longitudinally on the midline, gaining access to the peritoneal cavity, performed an abdominal washout clearing an only slightly turbid fluid, and changed the drape-gauze “sandwich” intraperitoneal dressing. As a consequence of an improvement of intra-abdominal edema and peritonitis under a constant low-tension made by the PP mesh, an initial relaxation of the muscular walls was obtained, allowing a partial re-approximation of the rectus sheaths. Hence, we removed a longitudinal segment of the polypropylene mesh and closed its defect with nylon running suture. The VAC system was finally reapplied.

One week later, the patient underwent another re-look laparotomy. The polypropylene mesh and the drape-gauze layer were ultimately removed, leaving the rectus fascia edges at approximately 15 cm apart at their maximum distance. We consulted our plastic and bowel surgery colleagues. Given the persisting risk of abdominal wound infection, decision was made not to use non-absorbable (polypropylene) mesh. Additionally, given the persisting relative frailty, invasiveness of dissection to the deeper abdominal wall planes was also avoided, choosing to perform anterior rather than posterior component separation. After completing abdominal wall anterior component separation from the subcutaneous tissue, an absorbable (Vicryl) inlay mesh could be accommodated, obtaining a reduction in the skin tension and limiting the deep abdominal wall dissection. We left two 15F Blake VAC suction drains in the neo-subcutaneous compartment. The skin was approached by Vicryl 3/0 dermal suture and 0 Nylon vertical mattress sutures, without evidence of skin ischemia.

Twenty-four hours after the last re-look the patient showed good medical condition, was extubated and sent back to surgical ward.

Nine days later, a trans anastomotic tube (Witzel) RX cholangiogram did not show any anastomotic leak or stricture, allowing the tube to be removed. The patient was discharged on day 17 from biliary reconstruction surgery able to walk without assistance. The postoperative course was then uncomplicated in the following three months (Dindo Clavien category 0).

**Fig. 5:**
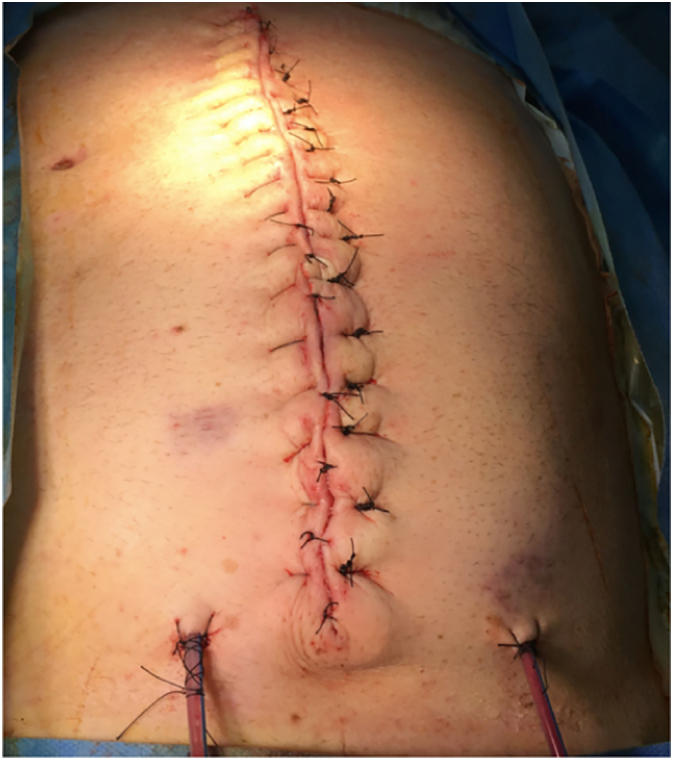
Final result.

An expected late incisional hernia was diagnosed about four months later and eventually repaired through posterior component separation. The midline closure was reinforced with a 5 × 20 cm longitudinal biological mesh (Permacol), obtaining a fully functional and aesthetic result (Fig. 5).

## 3 Timeline

Laparoscopic cholecystectomy was performed by an experienced general surgical team in a secondary Hospital.

On postoperative day 2, following emergency re-admission abdominal CT scan and ERCP revealed full CBD transection with biliary peritonitis. The patient was taken to theatre by the local surgical team for midline exploratory laparotomy, washout, and external drainage.

On post-operative day 11 after index cholecystectomy a wound dehiscence was locally treated by vacuum therapy dressing, eventually stopped due to full bowel herniation.

On postoperative day 14 after index cholecystectomy, at transfer arrival at our HPB service, a Roux-en-Y hepaticojejunostomy along with management of defect of abdominal wall with vacuum-assisted wound closure and mesh-mediated fascial traction (VAWCM) was performed by the on-call HPB consultant surgeon, with over a decade of general surgery experience and super-specialist HPB training.

On postoperative day 17 after index cholecystectomy, the patient underwent a re-look laparotomy with partial re-approximation of the rectus sheaths.

On postoperative day 24 after index cholecystectomy, the patient underwent another re-look laparotomy with complete re-approximation of the abdominal wall through extensive anterior component separation and placement of an absorbable mesh.

About four months after index cholecystectomy, an expected late incisional hernia was repaired through posterior component separation and biological mesh.

## 4 Discussion

The presented case warrants reporting as it presented with the very unusual combination of severe BDI, with unfavorable timing from the initial injury, with biliary peritonitis, and a fully dehisced long “burst” abdomen laparotomy, necessarily requiring immediate peritoneal clearance from sepsis.

Since its introduction in the late 1980s [26], [27] laparoscopic cholecystectomy has revolutionized the surgical approach to gallstone disease treatment. With the rapid spread of this technique, a reduction in minor complications such as wound infections, as well as shorter hospitalization, earlier recovery and return to normal activities have been witnessed [2]. However, a parallel increase in major bile duct injuries (BDI) has been reported in the literature since [28]. Hence, despite a progressive technical advancement in making laparoscopic cholecystectomy a safe procedure, the management of BDIs remains challenging [4]. BDIs are still burdened by mortality rates of up to 7% in complex injuries, and late complications can rarely result in end-stage liver disease [29].

The goal of BDI surgical repair is the restoration of the continuity of the injured bile duct. Roux-en-Y HJ is the most common surgical treatment of major BDI [15]. Many studies have measured complications' rates of up to 20–40% following HJ in the short-term. However, there has been a considerable decline in the operative mortality, with large series reporting no cases of perioperative deaths [30]. Common long-term complications of HJ include anastomotic stricture, recurrent cholangitis and secondary biliary cirrhosis [31]. The most feared complication is the anastomotic stricture, with an approximate incidence of 2%–25% [17], causing lower quality of life, morbidity and mortality [32], [33], [34]. Hence, the absence of biliary anastomotic stricture is the goal of a successful surgical management [35].

BDIs can be treated immediately in the absence of bile/fluid collections, or at least six weeks after the intra-abdominal infection has subsided (usually by means of percutaneous drainage), along with reduced inflammation [1], [12], [13], [36]. In our case, we decided to treat the BDI with HJ even though it was fourteen days after the injury. This was different from the ideal management suggested by the available evidence and the common practice. Our choice was made necessary by the diffuse peritonitis, which had led to the full wound dehiscence with evisceration.

However, the decision brought another challenge, making the complexity of the present case, started by the coexistence of two serious surgical indications, both potentially lethal, usually requiring different timing of repair to minimize their respective risks. A controlled BDI would have better required delayed ultimate repair, while diffuse peritonitis and burst abdomen required immediate laparotomy and complete repair.

We opted for an open abdomen (OA) management with temporary abdominal closure (TAC) in order to avoid abdominal compartment syndrome [37]. The OA technique was described for the first time by Mc Cosh more than a century ago [38]. However, this management for long time gained only very little popularity for the treatment of severe surgical conditions as its principles had been historically based on anatomical repairs with the objective of primary and definite organ salvage [39]. Eventually, over the last two decades the acknowledged importance of physiological homeostasis has pushed for developing the concept of damage-control surgery with special emphasis on the demand for open abdominal maintenance by laparostomy [40], [41].

Excessive visceral edema following a laparotomy for severe abdominal sepsis prevents successful tension-free fascial closure, and represents an indication for adopting OA. The main goal of OA is the closure of the fascial defect as early as it is clinically feasible without precipitating abdominal compartment syndrome [20]. The indications for OA management with TAC include severe intra-abdominal infections with peritonitis, bowel obstruction, pancreatitis, abdominal compartment-syndrome, control surgery and other strategies involving a planned re-laparotomy [37], [42]. However, OA can be associated with serious complications, such as fluid and protein loss, which must be carefully managed as they can produce nutritional deficiency and a catabolic state, loss of abdominal domain from fascial retraction, and eventually huge ventral hernia [43], [44].

Various additional techniques have been applied to the OA management of critically ill surgical patients since the 1980s (Table 1) [45], [46].

**Table 1:** Temporary abdominal closure techniques [45], [46].

	Technique	Description
1.	Simple packing	Non-adherent wet gauzes or hydrophilic dressings are positioned directly on top of the abdominal contents, without the use of any sutures.
2.	Bogota bag	A sterile irrigation bag is used to cover the abdominal viscera.
3	Mesh	An absorbable or non-absorbable prosthesis is sutured to approximate the fascial edges.
4.	Zipper	A mesh with a zipper is sutured between the fascial edges.
5.	Wittmann patch (artificial burr)	Two Velcro pieces are sutured to the fascial edges.
6.	Dynamic retention suture	Extraperitoneally placed large, non-absorbable sutures through all layers of the abdominal wall, including the skin. Sutures can be gradually tightened. May be combined with a NPWT system. Commercially available systems include ABRA Abdominal Wall Closure System (Canica Design).
7.	NPWT	A perforated plastic sheet is placed to cover the viscera and then a polyurethane sponge, or moist surgical towels/pads are placed on top, between the fascial edges. The final layer is constituted by an airtight dressing with a suction drain connected to a pump and fluid connection system.
8.	VAWCM	Modification of NPWT, using a mesh stitched to the fascial edges, which can be tightened at every NPWT system change.

Older static TAC techniques, e.g., Bogota bag or placement of a temporary mesh, do not facilitate closure and frequently result in large planned ventral hernias, concomitant morbidity and impaired quality of life [47].

The combination of a vacuum technique and mechanical approximation of the fascia seems to be the latest stage in the evolution of open abdomen treatment [48], [49]. In 2007 Petersson et al. first described the use of an alloplastic mesh in conjunction with a vacuum-assisted wound-closure technique called VAWCM (vacuum-assisted wound closure and mesh-mediated fascial traction) [23]. The objective of such a treatment is to achieve synergistic effects of edema reduction and fascial traction [50]. Negative pressure therapy (NPT) can increase local blood perfusion and nutrient delivery to the wound, as well as accelerate the growth of granulation tissues, and decrease wound bacterial presence. NPT has shown to reduce bowel edema, reduces mechanical stress to the wound and can accelerate cellular proliferation and angiogenesis and, by the principle of reverse tissue expansion in the wound, brings the wound edges together [51]. Finally, NPT has a positive effect on the treatment of abdominal-compartment syndrome [52].

Nutrition plays a key role during open abdomen management. Early enteral feedings should always be attempted in patients with an open abdomen because they promote gut-mediated immunity, maintain microbial diversity, increase intestinal blood flow, and attenuate the associated hyperdynamic stress response and immune suppression seen in these patients [53], [54], [55]. Early enteral feedings are associated with increased rates of primary fascial closure, lower fistula rates, and lower hospital charges [54].

## 5 Conclusion

BDI is a formidable complication of both open and laparoscopic cholecystectomy. In this case, BDI was associated with biliary peritonitis and abdominal evisceration, and forced an earlier operative management. We opted for a combined and immediate Roux-en-Y hepaticojejunostomy over a percutaneous external biliary transanastomotic tutor and partial drainage, as well as the contemporary management of the defect of abdominal wall with vacuum-assisted wound closure and mesh-mediated fascial traction (VAWCM). Such combination has proven safe and successful.

## Consent

Written informed consent was obtained from the patient for publication of this case report and accompanying images. Such written consent is included in the filed patient medical notes.

## Ethical approval

Not applicable.

## Funding

None.

## Guarantor

Dr. Roberto Valente.

## Research registration number

Not applicable.

## CRediT authorship contribution statement

Dr. Alfredo Torretta: analysis and interpretation of data, drafting the article,
Dr. Dimana Kaludova: data collection
Dr. Mayank Roy: data collection.
Dr. Satya Bhattacharya: treating surgical consultant.
Dr. Roberto Valente: mentor through process; treating surgical consultant; final approval of the version to be submitted.

## Declaration of competing interest

None.
